# From Blood to Outcome: Inflammatory Biomarkers in Rectal Cancer Surgery at a Romanian Tertiary Hospital

**DOI:** 10.3390/diseases13070218

**Published:** 2025-07-13

**Authors:** Georgiana Viorica Moise, Catalin Vladut Ionut Feier, Vasile Gaborean, Alaviana Monique Faur, Vladut Iosif Rus, Calin Muntean

**Affiliations:** 1 Department of Doctoral Studies, Victor Babes University of Medicine and Pharmacy, 300041 Timisoara, Romania; georgiana.moise@umft.ro; 2 Medical Oncology, “Pius Brinzeu” Clinical Emergency Hospital, 300723 Timişoara, Romania; 3 Abdominal Surgery and Phlebology Research Center, Victor Babeș University of Medicine and Pharmacy, 300041 Timisoara, Romania; 4 First Surgery Clinic, “Pius Brinzeu” Clinical Emergency Hospital, 300723 Timişoara, Romania; 5 Thoracic Surgery Research Center, “Victor Babeş” University of Medicine and Pharmacy Timişoara, Eftimie Murgu Square No. 2, 300041 Timişoara, Romania; 6 Department of Surgical Semiology, Faculty of Medicine, “Victor Babeş” University of Medicine and Pharmacy Timişoara, Eftimie Murgu Square No. 2, 300041 Timişoara, Romania; 7 Faculty of Medicine, “Victor Babeş” University of Medicine and Pharmacy Timişoara, 300041 Timişoara, Romania; alaviana.faur@student.umft.ro (A.M.F.); iosif.rus@student.umft.ro (V.I.R.); 8 Medical Informatics and Biostatistics, Department III-Functional Sciences, “Victor Babeş” University of Medicine and Pharmacy Timişoara, Eftimie Murgu Square No. 2, 300041 Timişoara, Romania; cmuntean@umft.ro

**Keywords:** rectal cancer, inflammatory biomarkers, AISI (aggregate index of systemic inflammation, SIRI (systemic inflammation response index), SII (systemic immune–inflammation index)

## Abstract

Background: Systemic inflammatory markers have emerged as accessible and reproducible tools for oncologic risk stratification, yet their prognostic value in rectal cancer remains incompletely defined, particularly in acute surgical settings. This study aimed to assess six inflammation-based indices—NLR, PLR, MLR, SII, SIRI, and AISI—in relation to tumor stage, recurrence, and outcomes among patients undergoing emergency versus elective resection for rectal cancer. Methods: We retrospectively evaluated 174 patients treated between 2018 and 2024. Pre-treatment blood counts were used to calculate inflammatory indices. Clinical and pathological parameters were correlated with biomarker levels using univariate and multivariate analyses. Results: Pre-treatment inflammation markers were significantly elevated in patients requiring emergency surgery (e.g., NLR: 3.34 vs. 2.4, *p* = 0.001; PLR: 204.1 vs. 137.8, *p* < 0.001; SII: 1008 vs. 693, *p* = 0.007), reflecting advanced tumor biology and immune activation. Notably, these patients also had higher rates of stage IV disease (*p* = 0.029) and permanent stoma (*p* = 0.002). Post-treatment, recurrence was paradoxically associated with significantly lower levels of SII (*p* = 0.021), AISI (*p* = 0.036), and PLR (*p* = 0.003), suggesting a potential role for immune exhaustion rather than hyperinflammation in early relapse. Conclusions: Inflammatory indices provide valuable insights into both tumor local invasion and host immune status in rectal cancer. Their integration into perioperative assessment could improve prognostication, particularly in emergency presentations. Post-treatment suppression of these markers may identify patients at high risk for recurrence despite initial curative intent.

## 1. Introduction

Rectal cancer (RC) continues to represent a major oncological burden worldwide, accounting for nearly one-third of all colorectal malignancies (CRC). Collectively, CRC ranks as the third most commonly diagnosed cancer and the second leading cause of cancer-related deaths globally [1]. According to GLOBOCAN 2020 data, over 1.9 million new cases of CRC and approximately 935,000 related deaths were reported worldwide. Rectal cancer (RC) accounted for a substantial portion of this burden, with over 310,000 deaths attributed to it [2]. Whereas countries with well-established healthcare infrastructures (USA, Germany, France) have significantly reduced mortality through organized CRC screening programs and early diagnostic strategies, much of Central and Eastern Europe continues to struggle with delayed detection. Structural limitations, insufficient screening coverage, and low public awareness contribute to a persistently high rate of advanced-stage diagnoses in this region [1].

Within Romania, epidemiological trends mirror this regional challenge. Recent data underscore a sustained rise in RC incidence, particularly among men aged over 60, with an alarming proportion of patients diagnosed in advanced stages of the disease [3]. These observations reflect an ongoing need for earlier detection and more refined prognostic stratification.

Despite significant advancements in neoadjuvant chemoradiotherapy (nCRT), total mesorectal excision, and multidisciplinary approaches, the prognosis of rectal cancer (RC) remains heterogeneous and heavily dependent on disease stage [4,5]. While five-year survival rates exceed 70% in cases of localized disease, they decline to below 15% in metastatic contexts, highlighting the critical need for prognostic tools that surpass the predictive capabilities of traditional staging systems [6,7].

Among the most promising adjunctive tools are inflammatory biomarkers derived from routine blood tests. Increasing evidence highlights systemic inflammation as a key driver of tumor progression, immunologic dysregulation, and resistance to treatment. Parameters such as the neutrophil-to-lymphocyte ratio (NLR), platelet-to-lymphocyte ratio (PLR), monocyte-to-lymphocyte ratio (MLR), systemic immune–inflammation index (SII), systemic inflammation response index (SIRI), and aggregate index of systemic inflammation (AISI) have drawn attention due to their accessibility, reproducibility, and capacity to reflect host–tumor interactions [8,9].

Several recent investigations have supported their prognostic relevance. Elevated pre-treatment values of these markers have been associated with diminished tumor regression following nCRT, lower disease-free survival (DFS), and reduced overall survival (OS). For instance, studies have shown that a baseline NLR of 2.3 or higher was predictive of poor oncologic response [8], while others identified post-treatment elevations in SII and SIRI as markers of suboptimal pathological regression [10,11,12].

However, the prognostic value of these markers is strongly influenced by the timing of measurement relative to disease progression or treatment. Measurements obtained prior to nCRT provide insight into tumor biology and systemic immune status, whereas those collected afterward may be affected by chemoradiotherapy-induced cytopenia or localized inflammation, potentially limiting their interpretability [13,14]. As noted by Xu et al. [15], evaluating how these markers evolve throughout treatment may offer stronger predictive insights than relying on static, single-timepoint values.

While most published studies group colon and rectal cancer patients together, the biological divergence between these entities—spanning genetic, epigenetic, and immune pathways—warrants disease-specific analysis. Our study addresses this gap by focusing exclusively on rectal cancer, thereby ensuring greater cohort homogeneity and more reliable interpretation of inflammation-based biomarkers [16,17].

In light of these considerations, our study explores the prognostic significance of six inflammation-based markers—namely NLR, PLR, MLR, SII, SIRI, and AISI—in a cohort of patients with rectal cancer who underwent either emergency surgery or elective resection. Both pre-treatment and post-treatment levels of these markers are analyzed in relation to tumor stage, recurrence, and the type of surgical intervention. By exploring these associations, we aim to provide a deeper understanding of the clinical utility of systemic inflammatory indices in the risk assessment and personalized management of rectal cancer.

## 2. Materials and Methods

For this retrospective study, medical records of patients diagnosed with rectal cancer and followed in the medical oncology outpatient clinic of the “Pius Brînzeu” County Emergency Clinical Hospital in Timișoara, Romania, were analyzed. Clinical data collected between 1 January 2018 and 1 October 2024 were included in the study.

This study employed clearly defined inclusion and exclusion criteria. Importantly, the study period overlapped with the COVID-19 pandemic, necessitating the exclusion of patients with a confirmed history of SARS-CoV-2 infection—either preceding or occurring during treatment—due to the virus’s potential to elicit a marked systemic inflammatory response that could confound biomarker analysis [18,19,20]. The analysis was restricted to patients who underwent surgical intervention for rectal cancer, with particular emphasis on the timing and context of surgery: emergency procedures performed without prior neoadjuvant chemoradiotherapy (nCRT) versus elective resections conducted following the completion of neoadjuvant treatment.

After meeting the inclusion criteria, data were collected for thorough statistical analysis and interpretation. Demographic variables such as gender, age, and place of residence (urban vs. rural) were recorded. Complete blood count parameters analyzed included

▪ Lymphocytes (Lym);
▪ Monocytes (Mon);
▪ Neutrophils (Neu);
▪ Platelets (Pla).

Based on these values, several inflammatory indices were calculated:

- NLR (neutrophil-to-lymphocyte ratio) = Neu/Lym;
- MLR (monocyte-to-lymphocyte ratio) = Mon/Lym;
- PLR (platelet-to-lymphocyte ratio) = Pla/Lym;
- AISI (aggregate index of systemic inflammation) = (Neu × Mon × Pla)/Lym;
- SIRI (systemic inflammation response index) = (Mon × Neu)/Lym;
- SII (systemic immune–inflammation index) = (Neu × Pla)/Lym.

A key aspect of the study was the timing of blood sample collection. Given the well-documented impact of chemoradiotherapy on the inflammatory status of patients [8], for patients undergoing emergency surgery, pre-treatment blood samples were collected at the time of hospital admission, prior to the urgent intervention. In elective cases, baseline blood tests were drawn before the initiation of neoadjuvant therapy. To preserve consistency, the final set of blood analyses included in the study corresponded to those obtained at the end of the therapeutic sequence—specifically, after the completion of both surgical and oncological treatment, and prior to hospital final discharge.

To assess comorbidities, the Charlson Comorbidity Index was applied. The study also considered the occurrence of disease recurrence or progression following the completion of both surgical and oncologic treatment. Histopathological evaluation of the resected specimens included analysis of tumor invasion (T), lymph node involvement (N), distant metastases (M), as well as the presence of lymphatic and perineural invasion, and overall disease staging. The presence of a permanent stoma was recorded. Additionally, data were collected on patients who were deceased at the time of retrospective analysis. Lastly, the interval between initial diagnosis and either recurrence or progression of the disease was examined. Owing to the retrospective nature of the study and occasional limitations in clinical documentation, disease recurrence and progression were recorded as a single outcome category, referred to as “relapse/progression” for consistency of analysis.

### Statistical Analysis

Statistical analysis and interpretation were carried out using IBM SPSS Statistics version 25 for Windows (IBM Corp., Armonk, NY, USA). The Shapiro–Wilk test was applied to assess the normality of continuous variables, with a significance threshold set at *p* < 0.05. Descriptive statistics, including measures of central tendency and dispersion, were used to summarize numerical data. For categorical variables, frequency distributions and percentages were reported to capture variation across groups. Group comparisons for normally distributed continuous variables were performed using Student’s *t*-test, while the Mann–Whitney U test was employed for non-normally distributed data. In cases involving more than two groups with normally distributed data, analysis of variance (ANOVA) was used. Categorical variables were compared using the Chi-squared test or Fisher’s exact test, depending on the distribution of expected frequencies. A *p*-value of less than 0.05 was considered statistically significant throughout all analyses.

## 3. Results

A total of 174 patients, aged between 31 and 82 years, were evaluated for this study. All patients were followed in the outpatient oncology department of the “Pius Brînzeu” County Emergency Clinical Hospital in Timișoara.

### 3.1. Emergency vs. Elective Surgery

Patients who underwent emergency surgical intervention were comparable to those who received elective surgery regarding sex distribution (67.3% vs. 66.1% male, *p* = 0.825), rural residence (53.8% vs. 49.4%, *p* = 0.446), and the proportion with a Charlson Comorbidity Index greater than 3 (54.9% vs. 62.4%, *p* = 0.186). However, a statistically significant difference in age was observed, with patients in the emergency surgery group being older on average than those treated electively (64.87 ± 8.5 vs. 61.71 ± 8.96 years, *p* = 0.002).

Patients who required emergency surgical intervention presented with more advanced stages of disease. Tumor-related factors such as depth of invasion (T), lymph node involvement (N), and presence of metastases (M) were significantly more severe in this group compared to patients who underwent elective surgery following neoadjuvant treatment. The distribution of these parameters is detailed in Table 1.

There was a significantly higher rate of permanent stoma among patients who underwent emergency surgery, with 33 individuals (63.46%) requiring a permanent stoma, compared to 47 patients (38.52%) in the elective surgery group (*p* = 0.002).

Patients who experienced complications from rectal cancer and required emergency surgery prior to neoadjuvant treatment had significantly lower lymphocyte counts (*p* < 0.001) and markedly higher levels of the inflammatory markers investigated. The results and their variations are presented in Table 2.

### 3.2. Deceased vs. Surviving Patients

Over the course of the study, 11 patients passed away. Among those who underwent emergency surgery, five deaths were recorded (9.6%), while six deaths occurred in the group that received neoadjuvant treatment (4.9%). The difference in proportions was not statistically significant (*p* = 0.244).

Patients who died during follow-up were similar to survivors in terms of age (61.0 ± 10.44 vs. 61.75 ± 8.8 years, *p* = 0.787), gender distribution (63.6% vs. 66.3% male, *p* = 0.859), and rural residence (63.6% vs. 48.5%, *p* = 0.330). Although a higher percentage of deceased patients had a Charlson Comorbidity Index greater than 3 (81.8% vs. 61.1%), the difference did not reach statistical significance (*p* = 0.170).

The variation in investigated parameters between deceased and surviving patients is presented in Table 3.

A significantly higher proportion of patients who died had a permanent stoma compared to those who survived (63.6% vs. 44.78%, *p* = 0.045), implicitly reflecting a lower rate of anastomosis in this subgroup.

Analysis of inflammatory marker variations revealed statistically significant differences in NLR and SII values among deceased patients. The results are presented in Table 4.

### 3.3. Regional Disease

Patients with regional stage disease, characterized by serosal invasion (pT = 4), were demographically similar to those without serosa involvement. However, neutrophil counts were significantly higher in the regional stage group (6.22 ± 3.72 vs. 5.06 ± 1.66, *p* = 0.032). Additionally, three of the evaluated inflammatory ratios were significantly elevated in patients with serosal penetration compared to those without. The results are detailed in Table 5.

### 3.4. Recurrence/Progression

A total of 26 cases of recurrence or disease progression were identified. There was no significant age difference between patients with recurrence (59.96 ± 7.69 years) and those without (62.01 ± 9.09 years, *p* = 0.280), and gender distribution remained consistent with the overall cohort (69.2% male, *p* = 0.714).

The proportion of patients with a Charlson Comorbidity Index greater than 3 was significantly higher among those who experienced recurrence (84.6% vs. 58.5%, *p* = 0.011).

In the non-recurrence group, stage III was the most common (48%), followed by stage II (24.3%), stage I (23%), and stage IV (4.7%). Conversely, in the recurrence/progression group, stage IV was most frequent (38.5%), followed by stage II (26.9%), stage III (19.2%), and stage I (15.4%). The distribution difference between the two groups was statistically significant (*p* < 0.001).

Among pre-treatment inflammatory markers, most showed no significant differences, with the exception of the systemic immune–inflammation index (SII), which was significantly higher in patients without recurrence (831.67 ± 635.3 vs. 491.38 ± 172.38, *p* = 0.036).

The mean duration from diagnosis to recurrence or progression was 623.69 ± 351.96 days. This interval was slightly shorter among patients who died (571 ± 304.6 days vs. 639.5 ± 370.73 days, *p* = 0.685).

At the conclusion of oncologic and surgical treatment, a final blood sample was analyzed. Among patients who later experienced recurrence or progression, significant post-treatment differences were observed in neutrophil count as well as in the PLR, AISI, and SIRI indices. The detailed results are presented in Table 6.

## 4. Discussion

In our cohort of 174 patients with rectal cancer, approximately 30% required emergency surgical intervention, most frequently due to obstructive or hemorrhagic complications. While the emergency and elective surgery groups were comparable in terms of gender distribution, rural residency, and comorbidity burden, patients undergoing emergency procedures were significantly older (mean age: 64.87 vs. 61.71 years, *p* = 0.002). This age disparity underscores the impact of delayed diagnosis among elderly individuals, which is associated with increased surgical risk and less favorable clinical outcomes [21]. This age gap is also reported in population-level analyses such as Araghi et al. [22], which attribute delayed diagnosis in older individuals to reduced access to screening and nonspecific early symptoms.

The emergency group exhibited markedly more advanced oncologic features. Stage III–IV disease was significantly more prevalent (*p* = 0.046), along with increased rates of pT4 invasion (*p* = 0.001) and nodal metastases (pN2: 38.4% vs. 9.8%, *p* < 0.001). These findings align with prior reports by Ma L et al. [23] and Sun J et al. [24], which highlight the association between omission of neoadjuvant therapy—often due to urgent surgical need—and greater tumor burden with lower resection completeness. A meta-analysis by Dossa et al. [25] further corroborates this, linking the absence of nCRT with poorer pathological staging and survival.

Distant metastases were also more frequent in the emergency group (*p* = 0.029), reinforcing the association between acute presentation and systemic disease, as noted by Lisanti et al. [26]. This pattern mirrors findings by Ding et al. [13], who reported strong links between late-stage surgery and M1 status in patients lacking preoperative assessment and systemic therapy. Pan et al. [27] similarly concluded that bypassing the multidisciplinary treatment pathway is associated with significantly worse oncologic outcomes in rectal cancer patients.

This unfavorable biological profile translated into more invasive surgical approaches and higher rates of permanent stoma formation (*p* = 0.002), mirroring data from Partl et al. [12], where emergent resections were associated with reduced chances for sphincter-preserving procedures. Nakamoto et al. [28] presented that permanent stoma rates were tightly linked to surgical urgency and the absence of neoadjuvant downstaging, particularly in tumors located <6 cm from the anal verge. Similar data are echoed in Western cohort studies, where early therapeutic planning significantly reduced the need for permanent stomas [29].

Our analysis identified significant hematologic differences between patients undergoing emergency versus elective surgery for rectal cancer, based on pre-treatment blood samples. Although total neutrophil and platelet counts were comparable between groups (*p* > 0.2), lymphocyte counts were markedly reduced in the emergency cohort (*p* < 0.001). This lymphopenia supports existing evidence that diminished lymphocyte-mediated immunity correlates with advanced disease and impaired antitumor response [17,30].

Consequently, all inflammation-based indices—NLR, PLR, MLR, SIRI, SII, and AISI—were significantly elevated in the emergency group, indicating a pronounced systemic inflammatory state. Notably, NLR (*p* = 0.001) and PLR (*p* < 0.001) were substantially higher, confirming their prognostic utility in identifying advanced-stage disease. These findings align with previous reports linking NLR >3.2 to deeper invasion (T4), higher nodal involvement (N2), and poor tumor regression in patients without neoadjuvant therapy [13,31].

Elevated SIRI (*p* < 0.001) and SII (*p* = 0.007) further underscore the interaction between tumor aggressiveness and systemic inflammation. Although derived from pre-treatment values, the inflammatory profile in emergency cases resembles that of treatment-resistant disease states, supporting observations from studies highlighting SII as a marker of residual burden and early recurrence [32,33].

AISI, a newer composite index integrating neutrophils, monocytes, and platelets relative to lymphocytes, was also significantly higher in the emergency group (*p* = 0.013). This marker offers a broader reflection of systemic inflammation. Nakamoto et al. [28] demonstrated that elevated preoperative AISI levels are associated with incomplete resection (R1–R2), early stoma formation, and reduced 3-year survival—all of which were more frequently observed in our emergency subgroup.

Out of 174 rectal cancer patients, 11 (6.3%) died, with higher but non-significant mortality in the emergency group (5 vs. 6, *p* = 0.244). Age, sex, and residence were similar between groups, pointing to tumor-related factors as a likely driver of outcomes. Although not significant, the higher prevalence of a Charlson Comorbidity Index >3 among deceased patients (81.8% vs. 61.1%) points toward greater systemic vulnerability—also emphasized by Lino Silva LS et al. [21] and Ma L et al. [23] as a determinant of poor outcomes.

Tumor staging had a clearer prognostic signal: stage IV and metastatic disease were significantly more frequent among deceased patients (*p* = 0.002), consistent with findings from the literature where distant spread at diagnosis correlated with inferior survival [13,26]. Permanent stoma rates were also higher in this group (*p* = 0.045), potentially reflecting advanced local invasion or technical constraints [12,28].

Notably, two inflammatory markers—NLR and SII—were significantly lower in patients who died (*p* = 0.009 and *p* = 0.026, respectively). While many studies associate high values with poor outcomes [31,34], our data suggest that, in certain cases, immune suppression rather than hyperactivation may indicate poor prognosis. This aligns with the hypothesis proposed by Martin-Carnicero A et al. [10], where low inflammatory markers reflected immune exhaustion, especially in advanced-stage patients.

In our cohort, patients with serosal invasion (pT4) exhibited significantly higher neutrophil counts compared to those without such invasion (*p* = 0.032), indicating an amplified systemic inflammatory response associated with deeper tumor infiltration. This was further reflected in elevated levels of NLR (*p* = 0.001), SII (*p* = 0.014), SIRI (*p* = 0.007), and AISI (*p* = 0.008).

These findings align with previous studies by Ding et al. [31], Tan Y et al. [34], and Partl et al. [12], which demonstrated that increased NLR and SII values are strongly associated with advanced T stage and suboptimal local control. Moreover, AISI has shown predictive value in identifying deeper tumor invasion [28]. Collectively, our results support the utility of these inflammation-based markers as non-invasive surrogates for detecting regionally advanced disease, with potential implications for preoperative risk assessment and surgical planning.

Interpretation of subgroup findings—particularly those concerning mortality and recurrence—should be approached with caution, as these analyses were conducted on relatively small patient subsets. Although the overall cohort was adequately powered for the main objectives, reduced numbers in these outcome-specific categories may limit statistical precision and the ability to draw firm conclusions. These preliminary trends are nonetheless valuable and highlight areas that warrant further investigation in larger, prospectively stratified studies.

Disease recurrence or progression occurred in 26 patients (14.9%), with no significant differences in age or gender distribution compared to non-relapsing patients. These findings are consistent with Ma L et al. [23] and Tan Y et al. [34], who also found no significant age or sex differences between relapsing and non-relapsing patients after rectal cancer surgery.

A more distinct pattern emerged regarding comorbidity burden. A CCI > 3 was significantly more common among patients who experienced recurrence (*p* = 0.011). This supports prior findings from Lino-Silva LS et al. [21] and Partl et al. [12], where higher CCI scores were linked to reduced immune resilience, treatment intolerance, and earlier relapse. Comorbid conditions may also limit the intensity or completion of neoadjuvant and adjuvant treatments, compromising overall oncologic control.

Among these patients, stage IV disease was most prevalent (38.5%), while stage III dominated in the non-recurrence group (48%). The significant difference (*p* < 0.001) confirms that baseline stage remains one of the most powerful predictors of recurrence, a fact consistently emphasized by Ding et al. [13], where metastatic disease at diagnosis carried a near four-fold increase in recurrence risk after curative-intent resection. These findings are also corroborated by large registries such as those presented by Araghi et al. [22], highlighting stage IV disease as a dominant determinant of poor long-term disease control in rectal cancer.

Interestingly, when evaluating systemic inflammatory markers measured before treatment, no statistically significant differences were observed between relapsing and non-relapsing patients, with the exception of SII, which was significantly lower in these patients (491.38 vs. 831.67, *p* = 0.036). This finding challenges the conventional paradigm, which links elevated inflammatory markers to poor outcomes. However, a similar paradoxical pattern was noted in the mortality subgroup of our cohort, where lower NLR and SII values were also associated with early death.

One possible explanation lies in the concept of tumor-induced immune suppression, particularly in advanced-stage or cachectic patients, where low systemic inflammatory markers may reflect immune exhaustion rather than a favorable status. This hypothesis was discussed by Martín-Carnicero et al. [10], where very low SII and AISI values were noted among patients with poor therapeutic response, despite being traditionally considered favorable markers. It is possible that patients with low pre-treatment SII represent an immunologically “silent” subgroup, where host–tumor interactions are skewed toward immune evasion rather than inflammation, contributing to disease progression.

The mean time to relapse or progression in our study was approximately 624 days, consistent with intervals reported in other studies [24,26] where the majority of relapses occurred within the first two years post-diagnosis. Although this interval was shorter in patients who subsequently died, the difference was not statistically significant. However, it suggests a trend toward faster progression in tumors with high-risk biological features or resistance to treatment.

After the completion of chemoradiotherapy and curative surgery, several systemic inflammatory markers remained significantly altered in patients who later experienced recurrence. Those with disease progression showed lower neutrophil counts (*p* = 0.016) and significantly reduced levels of PLR (*p* = 0.003), AISI (*p* = 0.036), SIRI (*p* = 0.017), and SII (*p* = 0.021) compared to patients with stable disease.

These findings, paradoxical at first glance, suggest that post-treatment inflammatory suppression may reflect an impaired immune recovery or persistent myelosuppression following multimodal oncologic therapy. Similar patterns were described in the literature where low post-CRT SII and SIRI values correlated with early relapse, potentially due to insufficient immune reconstitution [10,34]. Shevchenko I et al. also emphasized that post-treatment immune suppression, rather than active inflammation, may signal poor systemic control and allow micrometastatic escape [9].

Furthermore, Mi et al. [35] showed in a meta-analysis that dynamic changes, rather than absolute post-treatment levels, were more predictive of recurrence in rectal cancer patients, suggesting the importance of longitudinal monitoring rather than single-point interpretation. Therefore, the flat inflammatory profile observed in relapsing patients may represent a form of post-therapeutic immune exhaustion, underlining the need for biomarker-guided surveillance strategies. This phenomenon may reflect a combination of tumor-driven immunosuppression and treatment-induced immune dysfunction. Before treatment, sustained antigenic stimulation within the tumor microenvironment contributes to T-cell exhaustion—defined by reduced cytokine production, overexpression of inhibitory receptors (e.g., PD-1), and diminished cytotoxic activity. This state is reinforced by immunosuppressive cells such as MDSCs and regulatory T cells, and by cytokines including IL-10 and TGF-β [36,37].

Following oncologic therapies, particularly chemoradiotherapy and cytotoxic regimens like FOLFOX or FOLFIRI, additional factors such as lymphodepletion, impaired hematopoietic recovery, and skewed myeloid-to-lymphoid ratios may further compromise immune competence [38,39,40]. These effects can persist beyond treatment, contributing to a blunted systemic inflammatory response. In some patients, this may manifest as deceptively low levels of inflammatory biomarkers, masking an impaired ability to mount effective immune surveillance [41,42].

### Study Limitations

Although our findings contribute meaningful insights, they must be interpreted with caution due to several limitations. The retrospective design limited our ability to control for confounding variables. Important clinical data such as smoking status, chronic inflammatory conditions, obesity, diabetes, genetic syndromes, and concurrent medication use were not uniformly available, potentially influencing inflammatory marker levels and outcomes. Tumor staging accuracy—especially for T and M components—may have been affected by variability in imaging techniques and interpretation, despite adherence to standard protocols. This could have introduced misclassification bias, impacting the predictive analysis of inflammation-based indices. Moreover, our study relied on static measurements of inflammatory markers, rather than tracking changes over time. Evaluating biomarker dynamics could offer deeper insights into treatment response and disease evolution. Although the Charlson Comorbidity Index was employed to account for baseline comorbidities, it may not fully reflect the influence of nutritional status—such as cachexia, hypoalbuminemia, or unintended weight loss—which are known to modulate systemic inflammation. Lastly, although the overall sample size was reasonable, subgroup analyses (e.g., mortality, recurrence) involved smaller patient numbers, limiting statistical power. These results should therefore be interpreted as exploratory, and further validation in larger, prospective cohorts is warranted. Despite these constraints, the study highlights the potential role of hematologic inflammatory markers as accessible, low-cost prognostic tools in rectal cancer. Prospective validation in larger, standardized cohorts remains essential.

## 5. Conclusions

This study highlights the prognostic relevance of systemic inflammation markers in rectal cancer. Elevated values of NLR, PLR, AISI, SIRI, and SII were significantly associated with more advanced disease stages, higher permanent stoma rates, and poorer surgical outcomes, especially in patients presenting without the benefit of neoadjuvant therapy. These indices reflect both tumor biology and host immune response, offering a practical and accessible tool for risk stratification.

Unexpectedly, lower post-treatment inflammatory indices such as SII and AISI were associated with recurrence and mortality in certain subgroups, suggesting that immune suppression may, paradoxically, predict worse outcomes in advanced-stage patients. This duality underscores the need for longitudinal monitoring and context-specific interpretation of inflammatory markers. Our findings support the integration of such biomarkers into preoperative evaluation algorithms and long-term surveillance protocols in rectal cancer care.

## Figures and Tables

**Table 1:** Key parameters.

Characteristic	All, n = 174	Emergency = 52	Elective, n = 122	*p*
Stage				0.046
I	38 (21.8%)	6 (11.5%)	32 (26.2%)	
II	43 (24.7%)	13 (25%)	30 (24.6%)	
III	76 (43.7%)	24 (46.2%)	52 (42.6%)	
IV	17 (9.8%)	9 (17.3%)	8 (6.6%)	
Lymphatic invasion	43 (25.6%)	17 (35.4%)	26 (21.7%)	0.065
Perineural invasion	40 (23.8%)	14 (29.2%)	26 (21.7%)	0.303
pT				0.001
1	6 (3.4%)	4 (7.7%)	2 (1.6%)	
2	41 (23.6%)	3 (5.8%)	38 (31.1%)	
3	101 (58%)	33 (63.5%)	68 (55.7%)	
4	26 (14.9%)	12 (23.1%)	14 (11.5%)	
pN				<0.001
0	86 (49.4%)	20 (38.5%)	66 (54.1%)	
1	56 (32.2%)	12 (23.1%)	44 (36.1%)	
2	32 (18.3%)	20 (38.4%)	12 (9.8%)	
pM	17 (9.8%)	9 (17.3%)	8 (6.6%)	0.029

Chi-squared or Fisher’s exact test was used to compare qualitative data.

**Table 2:** Variation in inflammatory ratios (Mean ± SD).

Marker	Emergency Surgery, n = 52	Elected Surgery, n = 122	*p*
Lymphocytes	1650 ± 724	2249 ± 766	<0.001
Monocytes	585 ± 268	653 ± 231	0.161
Platelets	295,000 ± 97,991	279,552 ± 105,126	0.441
Neutrophils	5623 ± 3175	5086 ± 1516	0.209
NLR	3.34 ± 2.4	2.4 ± 0.79	0.001
MLR	0.41 ± 0.29	0.3 ± 0.09	0.002
PLR	204.13 ± 96.71	137.83 ± 64.25	<0.001
AISI	717.93 ± 825.78	462.48 ± 314.08	0.013
SIRI	2.49 ± 2.18	1.56 ± 0.68	<0.001
SII	1008.42 ± 900.44	693.47 ± 392.82	0.007

Student’s *t*-test was used to compare independent groups

**Table 3:** Cancer stage and tumor characteristics.

Characteristic	All, n = 174	Dead, n = 11	Alive, n = 163	*p*
Stage				0.023
I	38 (21.8%)	2 (18.2%)	36 (22.1%)	
II	43 (24.7%)	2 (18.2%)	41 (25.2%)	
III	76 (43.7%)	3 (27.3%)	73 (44.8%)	
IV	17 (9.8%)	4 (23.5%)	13 (8%)	
Lymphatic invasion	43 (25.6%)	5 (45.5%)	38 (24.2%)	0.118
Perineural invasion	40 (23.8%)	4 (36.4%)	36 (23.9%)	0.312
pT				0.874
1	6 (3.4%)	0	6 (3.7%)	
2	41 (23.6%)	2 (18.2%)	39 (23.9%)	
3	101 (58%)	7 (63.6%)	94 (57.7%)	
4	26 (14.9%)	2 (18.2%)	24 (14.7%)	
pN				0.201
0	86 (49.4%)	6 (54.5%)	80 (49.1%)	
1	56 (32.2%)	1 (9.1%)	55 (33.7%)	
2	32 (18.3%)	4 (36.4%)	28 (17.2%)	
pM	17 (9.8%)	4 (36.4%)	13 (8%)	0.002

Chi-squared or Fisher’s exact test was used to compare qualitative data.

**Table 4:** Variation in inflammatory ratios (Mean ± SD).

Marker	Dead, n = 11	Alive, n = 163	*p*
Lymphocytes	2128 ± 779	2063 ± 805	0.808
Monocytes	582 ± 362	637 ± 239	0.5
Platelets	248,800 ± 47,779	287,264 ± 105,868	0.258
Neutrophils	4297 ± 1412	5301 ± 2141	0.195
NLR	1.48 ± 0.88	2.79 ± 1.53	0.009
MLR	0.26 ± 0.13	0.34 ± 0.18	0.173
PLR	128.93 ± 47.06	160.32 ± 83.04	0.242
AISI	288.94 ± 258.96	561.12 ± 545.81	0.122
SIRI	1.38 ± 0.84	1.85 ± 1.37	0.345
SII	380.04 ± 261.46	823.66 ± 615.94	0.026

Mann–Whitney test was used to compare the two independent groups.

**Table 5:** Inflammation status in regional vs. non-regional disease (Mean ± SD).

Ratio	Regional Stage of Disease	Non-Regional Stage of Disease	*p*-Value
NLR	3.79 ± 2.8	2.5 ± 1.12	0.001
AISI	846.6 ± 1087.02	488.34 ± 354.9	0.008
SII	1111.06 ± 112.66	734.69 ± 457.61	0.014
SIRI	2.60 ± 2.81	1.68 ± 0.83	0.007

Chi-squared test was used to compare qualitative data.

**Table 6:** Variation in inflammatory markers after completion of treatment (Mean ± SD).

Marker	Relapse/Progression, n = 26	No Relapse, n = 148	*p*
Lymphocytes	1345 ± 840	882 ± 388	0.001
Monocytes	467 ± 214	471 ± 215	0.938
Platelets	202860 ± 82618	226920 ± 68539	0.234
Neutrophils	3050 ± 1273	5622 ± 3577	0.016
NLR	3.18 ± 3	7.11 ± 8.26	0.082
MLR	0.44 ± 0.25	0.64 ± 0.45	0.117
PLR	177.75 ± 58.65	310.56 ± 162.07	0.003
AISI	244.5 ± 273.14	907.86 ± 1160.9	0.036
SIRI	1.38 ± 1.41	4.36 ± 4.52	0.017
SII	458.08 ± 395.09	1669.9 ± 1918.62	0.021

Student’s *t*-test and Mann–Whitney tests were used to compare independent groups.

## Data Availability

The datasets used and/or analyzed during the current study are available from the corresponding author upon reasonable request.
